# Differentially Expressed lncRNAs Related to the Development of Abdominal Fat in Gushi Chickens and Their Interaction Regulatory Network

**DOI:** 10.3389/fgene.2021.802857

**Published:** 2021-12-24

**Authors:** Bin Zhai, Yinli Zhao, Shengxin Fan, Pengtao Yuan, Hongtai Li, Shuaihao Li, Yuanfang Li, Yanhua Zhang, Hetian Huang, Hong Li, Xiangtao Kang, Guoxi Li

**Affiliations:** ^1^ College of Animal Science and Technology, Henan Agricultural University, Zhengzhou, China; ^2^ College of Biological Engineering, Henan University of Technology, Zhengzhou, China; ^3^ Henan Key Laboratory for Innovation and Utilization of Chicken Germplasm Resources, Zhengzhou, China

**Keywords:** gushi chicken, abdominal fat, long noncoding RNA, regulatory network, ceRNA

## Abstract

Chickens are one of the most important sources of meat worldwide, and the growth status of abdominal fat is closely related to production efficiency. Long noncoding RNAs (lncRNAs) play an important role in lipid metabolism and deposition regulation. However, research on the expression profile of lncRNAs related to the development of abdominal fat in chickens after hatching and their interaction regulatory networks is still lacking. To characterize the lncRNA expression profile during the development of chicken abdominal fat, abdominal adipose tissues from 6-, 14-, 22-, and 30-week-old Chinese Gushi chickens were herein used to construct 12 cDNA libraries, and a total of 3,827 new lncRNAs and 5,466 previously annotated lncRNAs were revealed. At the same time, based on the comparative analysis of five combinations, 276 differentially expressed lncRNAs (DE-lncRNAs) were screened. Functional enrichment analysis showed that the predicted target genes of these DE-lncRNAs were significantly enriched in pathways related to the posttranscriptional regulation of gene expression, negative regulation of cell proliferation, cell adhesion and other biological processes, glycosphingolipid biosynthesis, PPAR signaling, fatty acid degradation, fatty acid synthesis and others. In addition, association analysis of the lncRNA transcriptome profile was performed, and DE-lncRNA-related lncRNA-mRNA, lncRNA-miRNA and lncRNA-miRNA-mRNA interaction regulatory networks were constructed. The results showed that DE-lncRNA formed a complex network with PPAR pathway components, including *PPARD*, *ACOX1*, *ADIPOQ*, *CPT1A*, *FABP5*, *ASBG2*, *LPL*, *PLIN2* and related miRNAs, including mir-200b-3p, mir-130b-3p, mir-215-5p, mir-122-5p, mir-223 and mir-125b-5p, and played an important regulatory role in biological processes such as lipid metabolism, adipocyte proliferation and differentiation. This study described the dynamic expression profile of lncRNAs in the abdominal fat of Gushi chickens for the first time and constructed the DE-lncRNA interaction regulatory network. The results expand the number of known lncRNAs in chicken abdominal fat and provide valuable resources for further elucidating the posttranscriptional regulatory mechanism of chicken abdominal fat development or deposition.

## 1 Introduction

Abdominal fat is the main type of fat tissue in chickens. In the past few decades, the growth rates and body weights of chickens have increased, resulting in the excessive deposition of body fat, especially abdominal fat, thereby reducing feed utilization and the rates of egg production, fertilization and hatching among other negative effects (Whitehead and Griffin, 1984; Triyuwanta et al., 1992; Knowles et al., 2008). Therefore, revealing the molecular mechanism of chicken abdominal fat development or deposition is of great significance for the development of genetic breeding strategies to improve abdominal fat deposition.

LncRNAs are noncoding RNAs greater than 200 nt in length that play important roles in many biological processes, such as the regulation of epigenetics, the cell cycle and differentiation (Fatica and Bozzoni, 2014). Many studies have confirmed that lncRNAs are also involved in the regulation of adipose tissue development or deposition-related biological processes (Grote and Herrmann, 2015; Kanduri, 2016; Van Solingen et al., 2018). For example, the lncRNA SRA can promote the expression of *C/EBPa*, *PPARγ*, *AdipoQ*, and *FABP4*, thereby promoting the differentiation of preadipocytes into mature adipocytes (Xu et al., 2010). LncRNA ADINR, which is transcribed approximately 450 bp upstream of the *C/EBPa* gene, can transcriptionally activate *C/EBPa* and promote adipogenesis (Xiao et al., 2015). In addition, lncRNAs can be used as competitive endogenous RNA (ceRNA) sponges to adsorb miRNAs and participate in the regulation of adipogenesis-related gene expression (Salmena et al., 2011; Tay et al., 2014). For example, the interaction of lncRNA IMFlnc1 and miR-199a-5p can upregulate *CAV-1* and promote adipogenesis in pig intramuscular adipocytes, and lncRNA Gm15290 promotes *PPARγ*induced fat deposition by adsorbing miR-27b (Liu et al., 2017; Zhang et al., 2019; Wang et al., 2020). These studies have increased our understanding of the molecular mechanisms regulating the development of animal adipose tissue, and lncRNAs have thus become new targets for elucidating the regulatory mechanism underlying the formation of animal adipose tissue-related traits. However, fewer studies were associated with chicken abdominal fat development (Muret et al., 2019).

Chickens are one of the most important agricultural animals (Becker et al., 1984), and a few studies have investigated lncRNAs related to fat formation. One study used high-throughput sequencing data from chicken preadipocytes and differentiated adipocytes to identify 3,881 lncRNAs and screened 235 DE-lncRNAs (Chen L. et al., 2019). Another study detected 27,023 lncRNAs from chicken abdominal preadipocytes and identified 1,098 DE-lncRNAs (Zhang et al., 2017). While these studies have facilitated our understanding of the roles of lncRNAs in the differentiation of chicken fat cells, more work is needed due to the following reasons. Firstly, these studies mainly focused on the differentiation stage of fat cells and not on the entire body, and studies on chickens after hatching are especially lacking. The expression profile of lncRNAs during adipose tissue development needs to be elucidated because the development of adipose tissue after hatching is closely related to chicken performance. Secondly, the timing characteristics of the lncRNA expression profile during chicken adipose tissue development after hatching are poorly understood. Finally, existing research is limited to only a few chicken breeds, while the characteristics of lncRNA expression profiles in the abdominal adipose tissues of many local chicken breeds with good meat quality have not been elucidated.

Gushi chicken is a famous local breed of meat and egg in China, which is often used as breeding material and breeding production. The variety reached sexual maturity at the age of 20 weeks, the peak of egg production was 27–35 weeks old, and the average annual egg production was 180. At the same time, this variety also has excellent meat quality characteristics, such as tender meat, delicious soup and so on, and it is often used as meat breeding. Abdominal fat deposition is closely related to meat quality characteristics, in order to reveal the molecular mechanism of lncRNA in the formation of meat quality traits of this variety, we herein elucidated the lncRNA expression profiles of the abdominal adipose tissues of Gushi chickens at 6, 14, 22, and 30 weeks of age and screened and identified DE-lncRNAs related to abdominal fat development. Thereafter, DE-lncRNA interaction regulatory networks were constructed. The results obtained expand the number of lncRNAs known to be related to chicken abdominal fat development and provide a basis for further research on the functional regulatory mechanisms of lncRNAs in chicken abdominal fat deposition for the identification of excellent local chicken resources in China.

## 2 Materials and Methods

### 2.1 Ethics Statement

Animal care in this study was performed in accordance with the Animal Experiment Management Regulations (Ministry of Science and Technology of China, 2004) approved by the Animal Care and Use Committee of Henan Agricultural University, China.

### 2.2 Animals and Sample Preparation

The Gushi chickens used as the experimental animals in this study were obtained from the Animal Center of Henan Agricultural University. A total of 200 one-day-old female Gushi chickens were raised in cages in the same environment under standard conditions used for the pure breeding conservation of Gushi chickens. In this study, three healthy chickens were randomly selected at 6, 14, 22, and 30 weeks of age, and twelve chickens were thus used in this study. The feeding method is the same as our previous research (Li Y. et al., 2021). The chickens were anesthetized by an intravenous injection of sodium pentobarbital (40 mg/kg) at a concentration of 0.2% into the wing vein. Under deep anesthesia, the chickens were euthanized by an intravenous injection of KCl (1–2 mg/kg), after which their abdominal adipose tissues were harvested, immediately frozen in liquid nitrogen and stored at −80°C until total RNA extraction.

### 2.3 Library Construction and Sequencing

Total RNA was extracted from the abdominal adipose tissues of Gushi chickens aged 6, 14, 22, and 30 weeks. The quality of RNA was evaluated by Microspectrophotometer and Agarose gel electrophoresis, and then used to construct cDNA library. An initial total amount of 3 μg of RNA was used for the construction of each library. First, ribosomal RNA was removed with the Epicentre Ribo-zero™ rRNA Removal Kit (Epicentre, Madison, Wisconsin, United States), and sequencing libraries were then generated using rRNA-depleted RNA with the NEBNext^®^ Ultra™ Directional RNA Library Prep Kit for Illumina^®^ (NEB, Ipswich, MA, United States). The products were then purified using a TruSeq RNASample Prep Kit v2 (New England Biolabs, Ipswich, MA, United States), and library quality was assessed on an Agilent Bioanalyzer 2100 system (Agilent Technologies, CA, United States). Finally, the libraries were sequenced using an Illumina HiSeq 2500 platform, and paired-end reads were generated.

### 2.4 Sequence Analysis and lncRNA Identification

Quality control of the sequencing data was conducted using fastp, and the reads of the adapter, the reads of multimers and the low-quality reads were removed to obtain clean reads (Chen et al., 2018). At the same time, the Q20, Q30, and GC contents of the pure data were calculated. All downstream analyses were based on high-quality clean data. Reference genome and gene model annotation files were downloaded from a genome website (ftp://ftp.ensembl.org/pub/release-104/fasta/gallus_gallus/dna/). An index of the reference genome was constructed using Hast2, and paired-end clean reads were aligned to the reference genome using Hast2 (Pertea et al., 2016). LncRNA expression was quantified using FeatureCounts software (Liao et al., 2014), and StringTie was used for the identification and quantification of transcripts from the aligned RNA-seq reads (Pertea et al., 2015). The transcripts obtained from the splicing of each sample were merged by using StringTie merge software, and transcripts with uncertain chain directions whose length did not exceed 200 nt were removed. The transcripts were then compared with the annotation of Ensembl version 104 using gffcompare (Pertea and Pertea, 2020), and the known coding protein transcripts were filtered out. Finally, three coding potential software programs, CPC2, PfamScan, and CNCI, were employed to screen for coding potential and thereby identify novel lncRNAs (Li et al., 2015; Kang et al., 2017; Luo et al., 2017).

### 2.5 Differential Expression Analysis

FeatureCounts software was used to calculate the number of lncRNAs in each sample, and StringTie was used to determine their expression levels based on FPKM values. Differentially expressed gene (DEG) analysis was performed using DESeq2 software V1.22.2. The thresholds of a log2 fold change (FC) value > 1.5 and a *q-value* < 0.05 indicated upregulated DE-lncRNAs, while a log2 (FC) value < −1.5 and a *q-value* < 0.05 indicated downregulated DE-lncRNAs (Love et al., 2014).

### 2.6 Cis- and Trans-Regulatory Target Gene Analysis

The cis- and trans-regulatory target genes of the DE-lncRNAs were predicted. Among them, the DEGs located approximately 100 kb upstream and downstream of the DE-lncRNAs were used as cis-acting target genes. In addition, a Pearson correlation coefficient (r) > 0.7 was used to predict the regulation of trans target genes by lncRNAs. Subsequently, gene ontology (GO) and KEGG pathway analyses of the predicted target genes of each group of DE-lncRNAs were performed using the Clusterprofiler package, and GO terms and KEGG pathways with a corrected *p*-value (*q* value) < 0.05 were considered to be significantly enriched (Yu et al., 2012).

### 2.7 Association Analysis and Interaction Network Construction

The transcriptome (PRJNA551368) and small RNA (PRJNA528858) library were constructed using the same RNA samples collected from the abdominal adipose tissues of Gushi chickens at the four developmental stages in our previous research. The association analysis of DE-lncRNAs was performed using these data (Chen Y. et al., 2019). miRanda and TargetScan software were used to predict the target interaction relationships between DE-lncRNAs and miRNAs (Agarwal et al., 2015). The differentially expressed lncRNA-miRNA-mRNA pairs were identified by Pearson’s correlation analysis. Combined with functional annotation, the lncRNA-miRNA mRNA pairs associated with lipid metabolism were selected, and Cytoscape software was used to construct their interaction network (Shannon et al., 2003).

### 2.8 Quantitative Real-Time PCR Analysis

To verify the accuracy of the data obtained by high-throughput sequencing, four lncRNAs were randomly selected, and the results were confirmed by qRT-PCR for nine randomly selected lncRNAs. The RNAs were reverse transcribed into cDNA with HiScript II Q RT SuperMix for qRT-PCR (+gDNA wiper) (Vazyme, Nanjing, China). qRT-PCR was performed on a LightCycler^®^ 96 qRT-PCR system (Roche, Basel, Switzerland) with ChamQ Universal SYBR qPCR Master Mix (Vazyme Biotech, Nanjing, China). *β-actin* were used as internal reference genes for mRNA. The relative expression levels were analyzed with the 2^−ΔΔCt^ method. Experiments were repeated at least three times, and all data are presented as the mean ± standard error of the mean. Data were analyzed and plotted with Prism software (GraphPad, San Diego, CA, United States). The qRT-PCR primer sequences are listed in Supplementary Table S1.

### 2.9 Data Analysis

The data were analyzed by single factor analysis of variance (ANOVA) with SPSS26.0. The difference significance test was carried out by Duncan multiple comparison. *p* < 0.05 was taken as the significant difference.

## 3 Results

### 3.1 Library Sequencing and lncRNA Identification

A total of 12 cDNA libraries were constructed using abdominal adipose tissues from Gushi chickens at four developmental stages. After sequencing and strict quality evaluation, each library contained 13.53–16.88 GB of clean bases, with the CG content ranging from 46.20 to 51.56%. Comparative analysis showed that 91.97–95.55% of the reads (83.08–90.16% of the number of species) were located in the reference genome. Details regarding the sequencing and quality evaluation of each library are presented in Supplementary Table S2.

**FIGURE 1:**
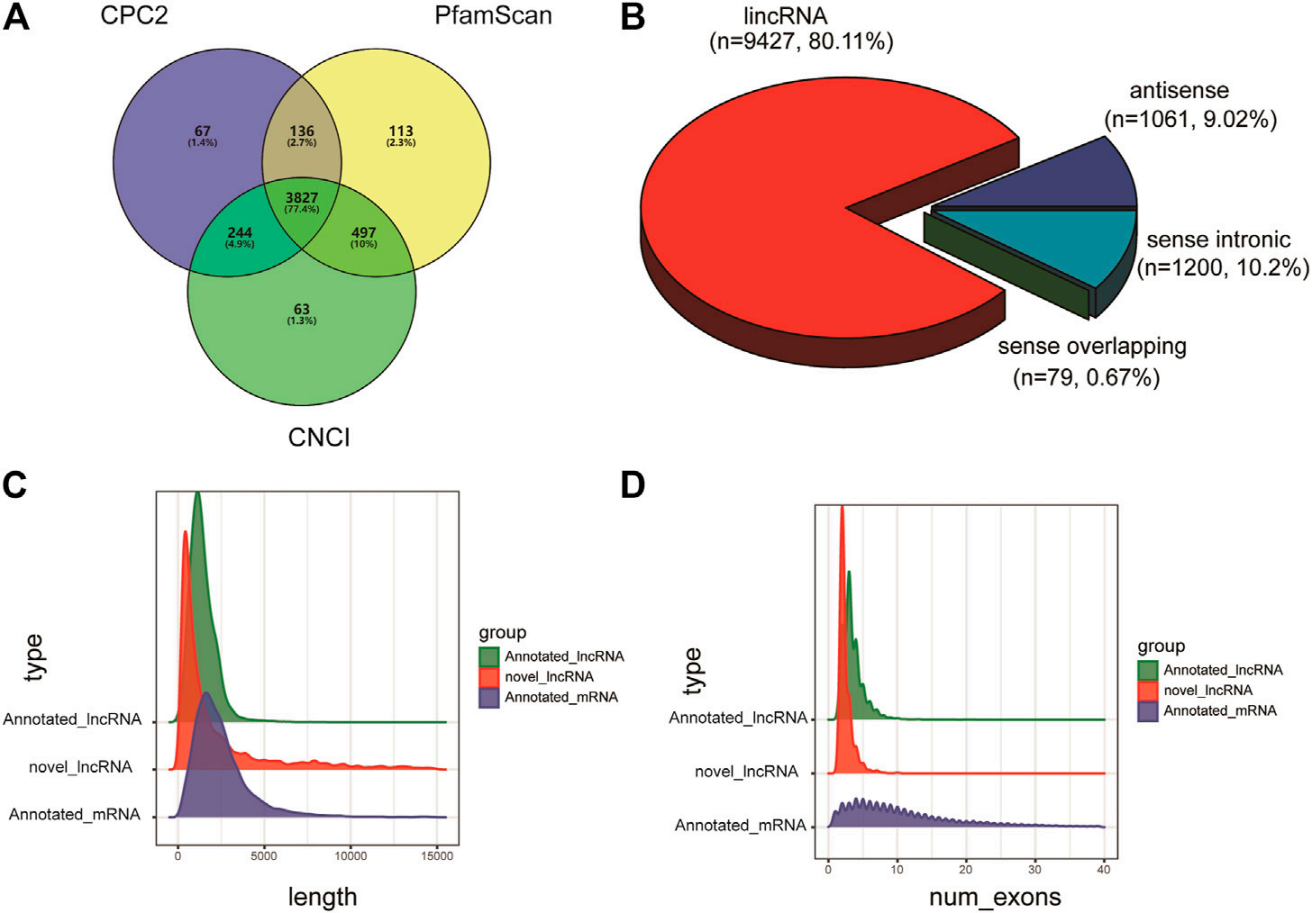
Library sequencing and lncRNA identification. **(A)** LncRNA identification through three databases, namely, Coding Potential Calculator (CPC), protein families database (PFAM) and Coding-Noncoding Index (CNCI). **(B)** The distribution of lncRNA transcript classification. **(C,D)** Distribution of transcript lengths, distribution of the number of exons per transcript (annotated lncRNA: green, and novel lncRNA: red, mRNA: blue).

StringTie merge software was used to merge the transcripts sequenced from each library, and the merged transcripts were then screened and annotated. Finally, a total of 5,466 known lncRNAs and 3,827 new lncRNAs were identified from the 12 libraries (Figure 1A). Among them, 9,427 lncRNA transcripts were located in the intergenic region, 1,061 lncRNA transcripts were located in introns, 1,200 lncRNA transcripts were antisense lncRNAs, and 79 lncRNAs were sense-strand overlapping transcripts (Figure 1B). In addition, structural comparison and analysis of the identified lncRNAs and mRNAs revealed that the lncRNAs were shorter and had fewer exons than the mRNAs (Figures 1C,D).

### 3.2 Characteristics of the Differentially Expressed lncRNAs

**FIGURE 2:**
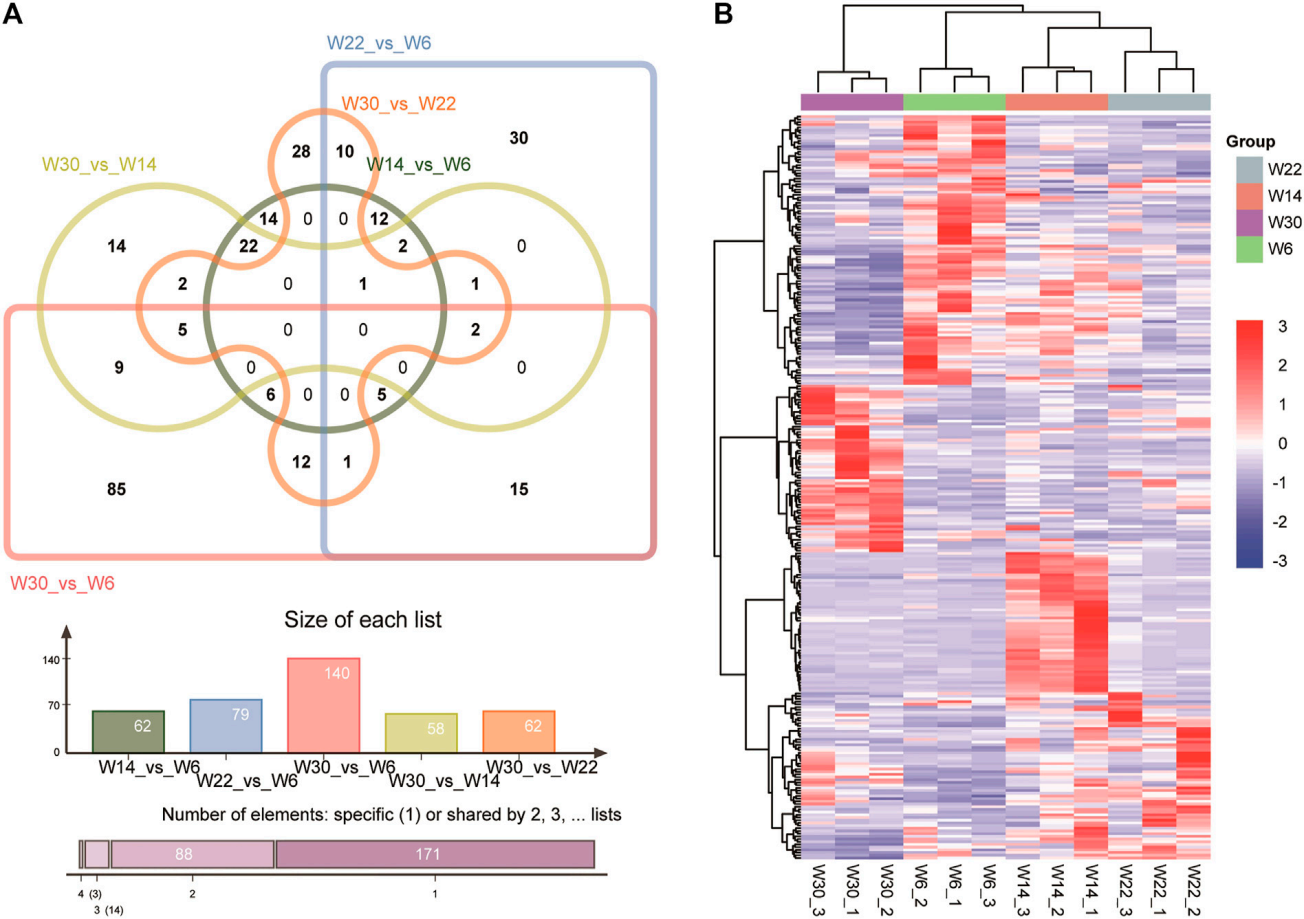
Characteristics of the differentially expressed lncRNAs. **(A)** Venn diagram of lncRNAs in the four developmental stages of Gushi chicken abdominal adipose tissue. **(B)** Heatmap showing DE-lncRNAs from different stages.

To gain insight into the key lncRNAs involved in chicken abdominal fat development, we analyzed the DE-lncRNAs (|fold change, FC| ≥ 1.5, *q*-value < 0.05) at four different developmental stages, namely, 6 weeks (W6), 14 weeks (W14), 22 weeks (W22), and 30 weeks (W30). Among the five different comparison groups, there were 62, 79, 140, 58, and 62 DE-lncRNAs in the W14 vs. W6, W22 vs. W6, W30 vs. W6, W30 vs. W14, and W30 vs. W22 comparison groups, respectively. However, DE-lncRNAs were not identified in the W22 vs. W14 comparison group. Venn diagram analysis showed that no DE-lncRNAs were common among the five comparison groups (Figure 2A).

**FIGURE 3:**
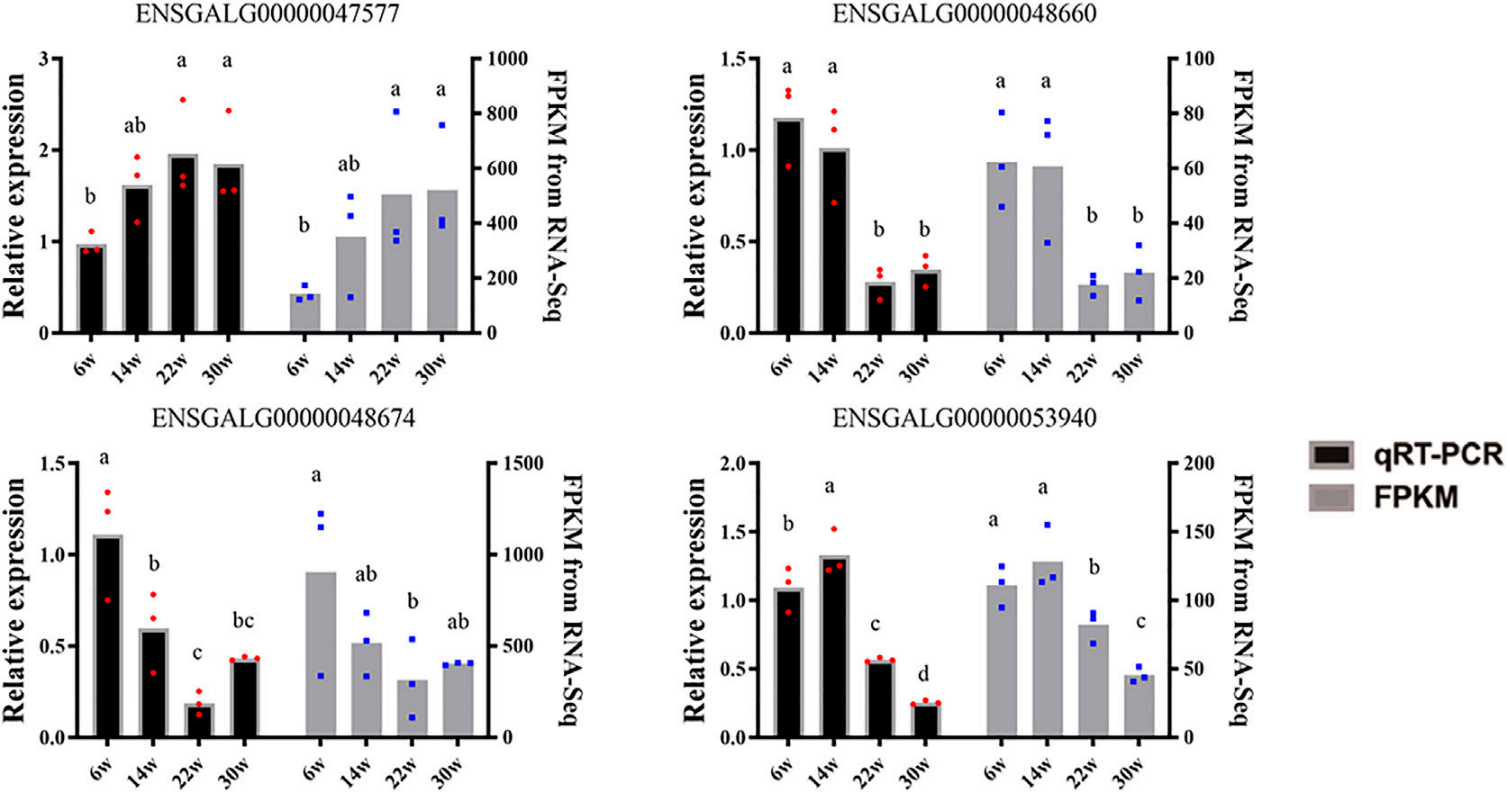
The qRT-PCR verification of DE-lncRNA. Black and gray represent the qRT-PCR results and the sequencing FPKM results, respectively. The data shoulder marks with the same letter or no letter indicate no significant difference (*p* > 0.05), while different letters indicate significant difference (*p* < 0.05).

LncRNAs MSTRG.16790 and MSTRG.13152 were the common DE-lncRNAs in four comparison groups, W22 vs. W6, W30 vs. W6, W30 vs. W14, and W30 vs. W22; lncRNA ENSGALG00000047342 was common among the W14 vs. W6, W22 vs. W6, W30 vs. W14, and W30 vs. W22 groups; and the other four combinations shared DE-lncRNAs. In particular, MSTRG.11907, MSTRG.2857, MSTRG.18130, MSTRG.9971, and ENSGALG00000053669 showed the same temporal expression characteristics in the W30 vs. W6, W30 vs. W14, and W30 vs. W22 comparisons. In addition, cluster analysis revealed the DE-lncRNAs in each group that clustered together, and the within-group differences were smaller than the between-group differences (Figure 2B). In particular, the 22- and 14-week-old clusters were closest, which was consistent with the result that no DE-lncRNAs were identified between 14 and 22 weeks of age. In addition, we randomly selected 4 DE-lncRNAs for qRT-PCR verification, and the results revealed an expression pattern similar that obtained by RNA-seq sequencing, which indicated that the RNA-seq sequencing data were authentic and reliable (Figure 3).

**FIGURE 4:**
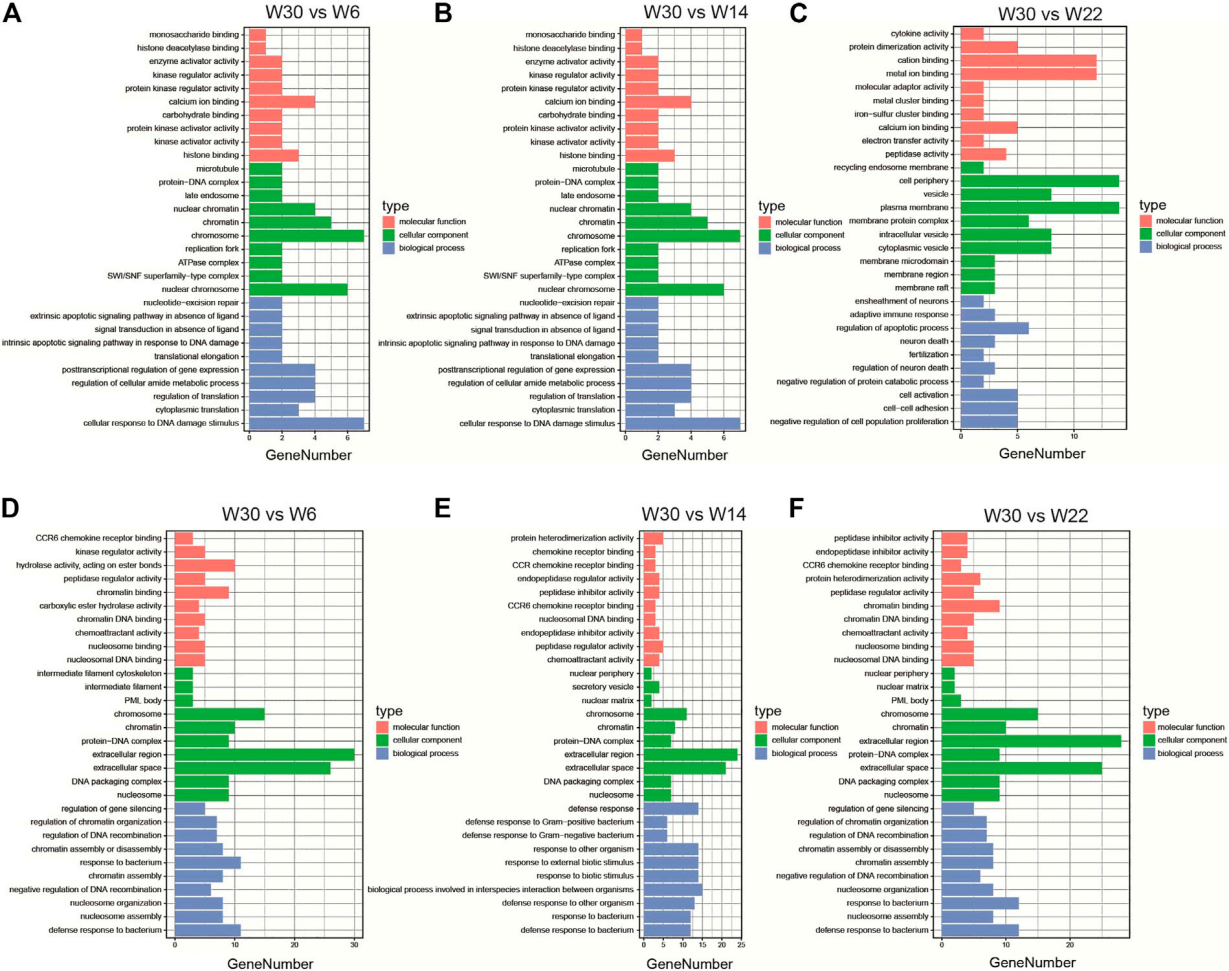
The enriched GO terms of the DE-lncRNA. The GO enrichment analysis results of DE-lncRNA cis **(A–C)** and trans **(D–F)** predicted target genes in the three comparison combinations. The figure shows the top 10 significantly enriched GO terms in the categories of biological processes, molecular functions, and cell composition.

**FIGURE 5:**
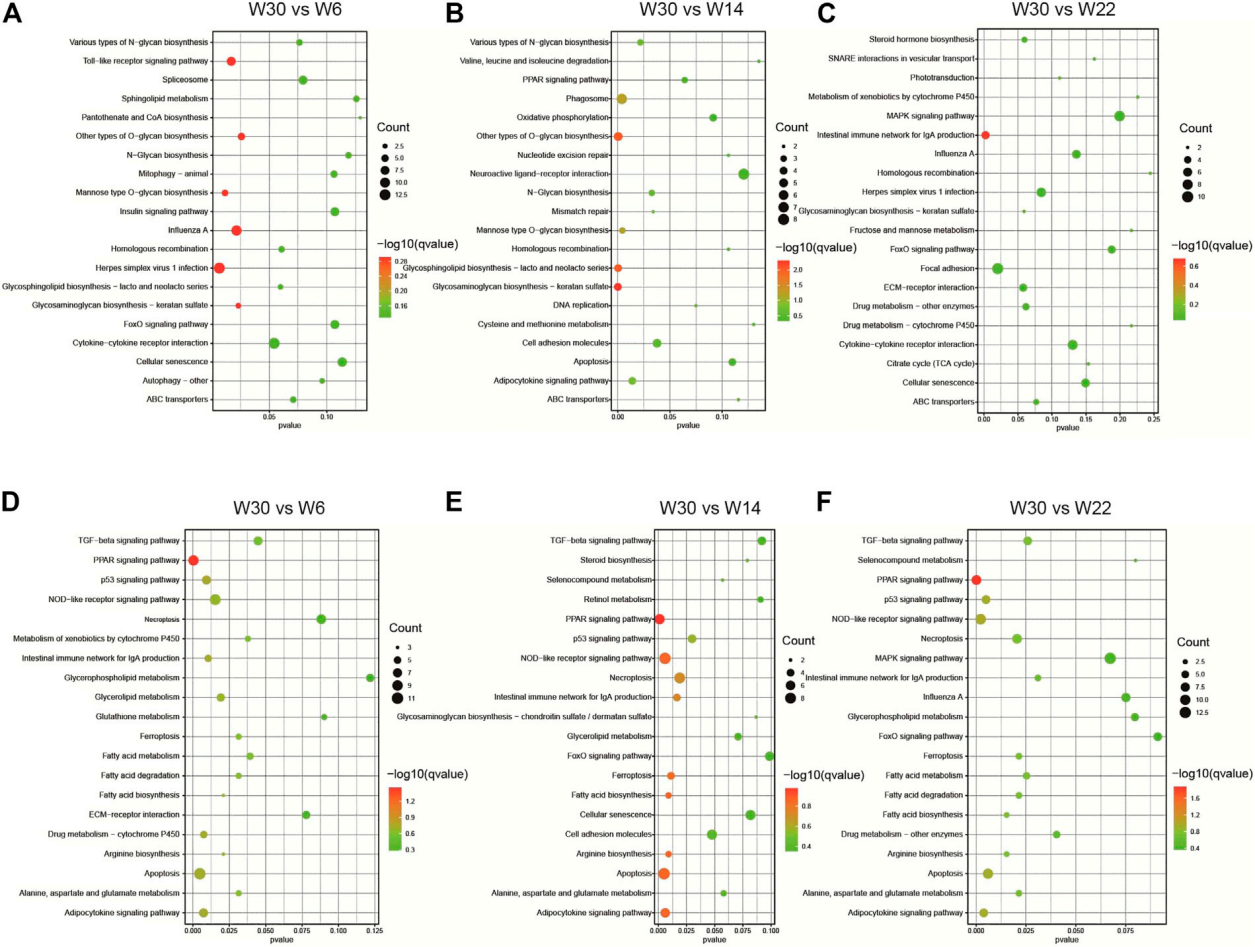
The enriched KEGG pathways of the DE-lncRNA. KEGG pathway enrichment analysis results of DE-lncRNA cis **(A–C)** and trans **(D–F)** predicted target genes in the three comparison combinations. The figure shows the top 20 KEGG pathways that are significantly enriched in each comparison.

To further elucidate the potential roles of the DE-lncRNAs in the development of abdominal fat in Gushi chickens, their cis- and trans-regulatory target genes were predicted, and functional enrichment analysis was performed. GO enrichment analysis results showed that the cis-regulated target genes showed significant enrichment for GO terms such as translation regulation, posttranscriptional regulation of gene expression, negative regulation of cell proliferation, and cell adhesion (Figures 4A–C). The trans-regulated target genes were significantly enriched for GO terms such as peptidase inhibitor activity and endopeptidase inhibitor activity (Figures 4D–F). In addition, KEGG pathway analysis revealed that the cis-regulated target genes showed significant enrichment for pathways related to glycosaminoglycan biosynthesis-keratan sulfate, glycosphingolipid biosynthesis-lactose and neolacto series, and other types of O-glycan biosynthesis (Figures 5A–C). The trans-regulated target genes showed significant enrichment for the PPAR signaling, fatty acid metabolism, fatty acid degradation, fatty acid biosynthesis, and adipocytokine signaling pathways (Figures 5D–F).

### 3.3 Interactions Between lncRNAs and mRNAs During Abdominal Fat Development

**FIGURE 6:**
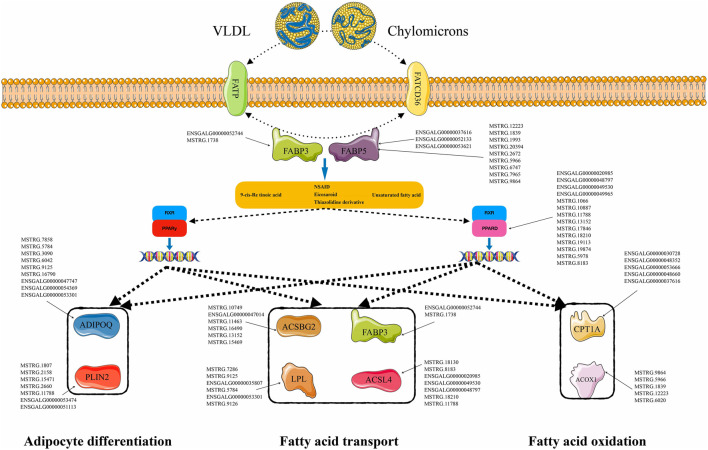
Differentially expressed lncRNA-mRNA interaction network related to PPAR signaling pathway. The interaction network between 150 DE-lncRNAs and 10 DE-mRNAs.

Based on the prediction results for the DE-lncRNA target genes, 9,207 cis-interaction regulatory relationships were observed between 136 DE-lncRNAs and 1,182 mRNAs, and 13,970 trans-interaction regulatory relationships were observed between 276 DE-lncRNAs and 559 mRNAs. Based on this, interaction regulatory networks between the DE-lncRNAs and their target genes was constructed. Many of these networks were related to lipid metabolism. In particular, the PPAR signaling pathway, which includes 10 genes, was significantly enriched in the W22 vs. W6, W30 vs. W6, and W30 vs. W22 comparison groups. In total, 275 lncRNA-mRNA interaction pairs were formed with 150 DE-lncRNAs (Figure 6), yielding a complex regulatory network. Among them, lncRNA ENSGALG00000037616 was shown to target the mRNAs of *ACOX1*, *ADIPOQ*, *CPT1A*, *FABP5* and other genes in the PPAR signaling pathway. These lncRNA-mRNA regulatory networks may play an important role in fatty acid metabolism and abdominal fat deposition in chickens.

### 3.4 Interactions Between lncRNAs and miRNAs During Abdominal Fat Development

**FIGURE 7:**
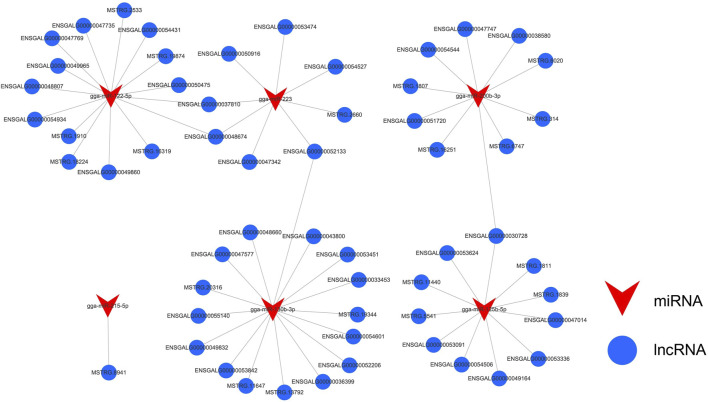
Differentially expressed miRNA-lncRNA interaction network related to lipid metabolism. Blue represents lncRNA and red represents miRNA. The interaction network contained 61 lncRNA-miRNA pairs formed by 57 DE-lncRNAs and 6 DE-miRNAs.

Based on the miRNA transcriptome profile data of abdominal fat samples from Gushi chickens at the four developmental stages, 51 differentially expressed miRNAs (DE-miRNAs) were identified, and the target relationships between the DE-lncRNAs and DE-miRNAs were predicted. In total, 4,058 lncRNA-miRNA interaction pairs (Supplementary Table S3) were observed between 272 DE-lncRNAs and 50 DE-miRNAs. Among these miRNAs, gga-miR-200b-3p, gga-miR-130b-3p, gga-miR-215-5p, gga-miR-122-5p, gga-miR-223, and gga-miR-125b-5p were previously shown to be related to lipid metabolism (Kim et al., 2013; Tao et al., 2016; Wei et al., 2017; Du et al., 2018; Xu et al., 2018; Li W. D. et al., 2019; He et al., 2019; Rodríguez et al., 2020; Li G. et al., 2021). To this end, we built a miRNA-lncRNA (FPKM > 10) interaction regulatory network based on these miRNAs (Figure 7).

### 3.5 Interactions Among lncRNAs, miRNAs and mRNAs During Abdominal Fat Development

**FIGURE 8:**
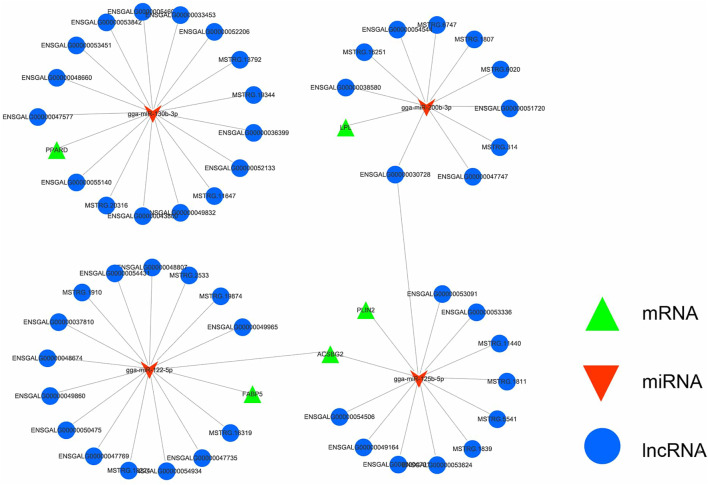
The lncRNA-miRNA-mRNA ceRNA network related to lipid metabolism. Green represents mRNA, red represents miRNA, and blue represents lncRNA. The interaction network contained 82 lncRNA-miRNA-mRNA pairs formed by 51 DE-lncRNAs, 4 DE-miRNAs and 5 DE-mRNAs.

Based on the whole-transcriptome data association analysis of abdominal adipose tissue samples from Gushi chickens at the four developmental stages, 1,740 differentially expressed lncRNA-miRNA-mRNA pairs (Supplementary Table S4) were screened to construct a ceRNA regulatory network related to abdominal fat development. In particular, we found a ceRNA regulatory network that was related to lipid metabolism (Figure 8), which contained 82 lncRNA-miRNA-mRNA pairs formed by 51 DE-lncRNAs, 4 DE-miRNAs and 5 DE-mRNAs. Meanwhile, we used lncLocator to predict the subcellular localization of 51 lncRNAs involved in the construction of ceRNAs (Cao et al., 2018). The results showed that 48 lncRNA predictions were located in the cytoplasm and another 4 lncRNAs were predicted to be located in Exosome and Cytosol. These results show that most of the lncRNAs we used to construct the ceRNA networks are located in cytoplasm (Supplementary Table S5). Among the components of this network, *ASBG2*, *LPL*, and *FABP5* are involved in fatty acid transport. They are related to three miRNAs (gga-miR-122-5p, ga-miR-125b-5p, and gga-miR-200b-3p), the latter two of which form 51 ceRNAs and 36 lncRNAs. In addition, *PLIN2* was related to adipocyte differentiation and forms 11 ceRNAs with gga-miRNA-125b-5p and 11 lncRNAs. In addition, *PPARD*, gga-miR-130b-3p and 16 lncRNAs also formed 16 ceRNAs.

## 4 Discussion

In poultry production, abdominal fat is often eliminated as a byproduct. Excessive abdominal fat deposits increase feed costs and affect chicken yield and quality. Therefore, revealing the molecular mechanism regulating abdominal fat deposition is of great significance for the selective breeding of chickens with optimal abdominal fat regulation. LncRNAs are widely involved in the regulation of biological processes such as gene expression, cell proliferation, cell differentiation, and cell apoptosis (Fatica and Bozzoni, 2014; Wei et al., 2015; Chen et al., 2017; He et al., 2019). Some recent studies have also indicated that lncRNAs play an important role in the formation of animal fat. For example, the antisense lncRNA PU.1 can promote the adipogenesis of pig preadipocytes by binding to PU.1 (Wei et al., 2015), and lncRNA U90926 can reduce the expression of *PPARγ2* or *PPARγ* and inhibit the differentiation of 3T3-L1 adipocytes (Chen et al., 2017). This indicates that lncRNAs can serve as new targets to analyze the formation of related traits in the adipose tissues of livestock and poultry. However, only a few studies have investigated the lncRNAs related to chicken adipose tissue development or deposition. Only these studies reported the identification of lncRNAs from chicken adipocytes (Zhang et al., 2017; Chen L. et al., 2019). In this study, we used high-throughput sequencing data and adopted three strategies to identify 5,397 known lncRNAs and 2,487 new lncRNAs in the abdominal adipose tissues of Gushi chickens, a local breed, at four developmental stages. These results enrich the data on lncRNAs in chicken abdominal fat and provide a valuable resource for further revealing the lncRNA regulatory mechanisms underlying chicken abdominal fat deposition.

In organisms, many lncRNAs have obvious temporal expression characteristics and play important roles in the regulation of specific biological processes. In this study, based on the lncRNA transcriptome profile of abdominal adipose tissue samples from Gushi chickens at four developmental stages, we identified a total of 276 significantly DE-lncRNAs from five comparative combinations. These DE-lncRNAs showed obvious tissue development stage specificity. Pathway enrichment analysis of the target genes predicted to be related to the DE-lncRNAs showed that in the W14 vs. W6 combination, the TGF-β signaling pathway and other signaling pathways related to cell proliferation were significantly enriched. In the W22 vs. W6 combination, the PPAR signaling pathway and other adipocyte signaling pathways related to differentiation or lipogenesis were significantly enriched. Finally, in the W30 vs. W22 combination, fatty acid metabolism, fatty acid synthesis, and other signaling pathways related to lipid metabolism were significantly enriched. These results indicate that the abdominal adipose tissues of Gushi chickens may be dominated by fat pads and adipocyte hyperplasia before 14 weeks of age, mainly by adipocyte hypertrophy at 14–22 weeks of age, and by adipocytes filled with fat after 22 weeks of age. This is consistent with the phenotypic changes in Gushi chicken abdominal fat. In the early stage, our research showed that the fat percentages (abdominal fat weight/live weight × 100%) of Gushi chickens at 6, 14, 22, and 30 weeks of age were 0.63 ± 0.42, 1.95 ± 1.08, 3.25 ± 1.26, and 2.96 ± 0.35, respectively. At 30 weeks, Gushi chicken had reached body maturity and was peak egg production. Due to the large energy consumption of egg production and less energy consumption for abdominal fat deposition, therefore, 14–22 weeks of age is an important stage for abdominal fat deposition in Gushi chickens. Therefore, the dynamic lncRNA expression profile revealed in this study truly reflects the molecular characteristics of Gushi chicken abdominal fat development.

Studies have shown that lncRNAs can directly interact with genes related to lipid metabolism or with miRNAs to regulate lipid metabolism. Through these regulatory methods, lncRNAs can increase the precision and complexity of posttranscriptional lipid metabolism regulation. To reveal this complex regulatory relationship of lncRNAs during the development of Gushi chicken abdominal fat, we constructed interactive regulatory networks between DE-lncRNAs and their target gene mRNAs and miRNAs. Among the genes in the constructed lncRNA-mRNA network, *ACSBG2*, *ACOX1*, *PPARD*, *ADIPOQ*, *CPT1A*, *ACSL4*, *PLIN2*, *LPL*, *FABP3*, *FABP5* and many other key genes related to lipid metabolism or fat deposition have been confirmed to play a role in fat formation in chickens (Nematbakhsh et al., 2021). For example, *PPARD* can enhance the conversion of chicken immature adipocytes to mature adipocytes by stimulating fatty acid oxidation (Sato et al., 2009; Guo et al., 2018), and *CPT1A* is a key factor that regulates fat deposition during chicken embryonic development and promotes chicken intramuscular growth by downregulating fatty acid oxidation and fat deposition (Qiu et al., 2017; Li S. et al., 2019). *PLIN2* participates in the regulation of fat deposition and is significantly related to the abdominal fat weight and percentage of abdominal fat in chickens (Zhao et al., 2007; Guo et al., 2018; Li et al., 2018). Finally, *FABP3* is significantly related to the intramuscular fat content in Beijing oil chickens and regulates intracellular fatty acid transport via a targeted interaction with lncRNA ENSGALG00000021686 (Li et al., 2008; Ye et al., 2010; Bi et al., 2020). Among the miRNAs in the constructed miRNA-lncRNA network, miR-200b-3p, miR-130b-3p, miR-215–5p, miR-122–5p, miR-223, and miR-125b-5p, among others, were previously demonstrated to be related to fat formation. Because the process by which lncRNAs are transcribed is highly similar to that of mRNAs, miRNAs can also target lncRNAs to enhance or inhibit their expression (Ballantyne et al., 2016)]. The abovementioned related studies show that the lncRNA-mRNA and miRNA-lncRNA interaction networks constructed herein are representative and can reflect the complexity of DE-lncRNA regulation in the development of Gushi chicken abdominal fat.

The regulatory mechanism of lncRNA is very complex. It has been found that lncRNA located in the cytoplasm can be used as a molecular sponge of miRNA to adsorb miRNA to remove the inhibition of miRNA on its target genes. (Salmena et al., 2011; Tay et al., 2014). Together with mRNAs, miRNAs and lncRNAs form a core network and jointly regulate gene expression at the transcription or posttranscription stage, thereby playing an important regulatory role in the formation of bodily traits. In this study, we constructed a DE-lncRNA related ceRNA network and predicted the subcellular localization of the selected lncRNAs. The results showed that most lncRNA were located in the cytoplasm. Through the construction of ceRNA networks, identified the core gene and miRNA components, such as *PPARD*, *PLIN2*, *FABP5*, *ACSBG2*, *LPL*, miR-200b-3p, miR-130b-3p, miR-215-5p, miR-122-5p, miR-223, and miR-125b-5p, most of which have been proven to be related to fat formation. Among them, *FABP5* is a member of the FABP gene family and participates in the absorption, metabolism, and transport of long-chain fatty acids (Chmurzyńska, 2006; Ye et al., 2010), miR-130 inhibits the differentiation of preadipocytes by inhibiting the expression of *PPARG* (Wei et al., 2017), and miR-125 regulates the proliferation and differentiation of preadipocytes (Du et al., 2018; Xu et al., 2018). These results indicate that DE-lncRNAs may play an important regulatory role in the development or deposition of abdominal fat in Gushi chickens in the form of ceRNAs.

In summary, we revealed the dynamic expression profile of lncRNAs related to abdominal fat development in Gushi chickens and identified 9,293 lncRNAs and 276 DE-lncRNAs. At the same time, a DE-lncRNA-related interaction regulation network was constructed, revealing that lncRNAs mainly affect lipid metabolism or deposition, adipocyte proliferation and differentiation and other biological processes through a complex interaction network, thereby regulating the development of chicken abdominal adipose tissue. These results provide a valuable resource for better understanding the posttranscriptional regulatory mechanism underlying chicken abdominal fat development and a basis and clues for additional research on the functional mechanism by which lncRNAs regulate chicken abdominal fat deposition.

## Data Availability

The datasets presented in this study can be found in online repositories. The names of the repository/repositories and accession number(s) can be found below: https://www.ncbi.nlm.nih.gov/, PRJNA528858 https://www.ncbi.nlm.nih.gov/, PRJNA551368.
