# Supplementary material for: Differentially Expressed lncRNAs Related to the Development of Abdominal Fat in Gushi Chickens and Their Interaction Regulatory Network
Source: Front Genet. 2021 Dec 24;12:802857. doi: 10.3389/fgene.2021.802857 (PMC8740130; doi:10.3389/fgene.2021.802857)
Supplement: Supplementary file 2 [file Table1.DOCX]

**Table S1. qRT-PCR Primers list.**

| **lncRNA** | **Transcripts Id** | **Primer Sequence（5’-3’）** | **AT(**°**C)** | **Length(bp)** |
| --- | --- | --- | --- | --- |
| ENSGALG00000047577 | ENSGALT00000100488 | F: TCACACTTTGTGCCTCTTCCT  R: GCTACTAACGCTTGCCTTCCA | 60 | 100 |
| ENSGALG00000048660 | ENSGALTI00000093135 | F: TGAGGCAAAACCACTTCCAGT  R: CCCCACCCTGACTCTGTAGT | 60 | 273 |
| ENSGALG00000048674 | ENSGALT00000092535 | F: GGGCAGGTGTAAACATTGGC  R: TCAGGATTGTGAAAGAAGTAGGAAC | 60 | 186 |
| ENSGALG00000053940 | ENSGALT00000105894 | F: GCTATGGAGAAGACCGGCTG  R: AAGCAGGGAACACCCACATC | 60 | 254 |
| *β-actin* |  | F: CACGGTATTGTCACCAACTG  R: ACAGCCTGGATGGCTACATA | 60 | 116 |

Abbreviation: Transcripts Id refers to the transcript id of lncRNA; AT refers to the annealing temperature; F and R refer to the forward and reverse primers, respectively; Length refers to the length of the product.
