# Supplementary material for: Differentially Expressed lncRNAs Related to the Development of Abdominal Fat in Gushi Chickens and Their Interaction Regulatory Network
Source: Front Genet. 2021 Dec 24;12:802857. doi: 10.3389/fgene.2021.802857 (PMC8740130; doi:10.3389/fgene.2021.802857)
Supplement: Supplementary file 3 [file Table2.DOCX]

**Table S2. Summary of draft reads of 12 cDNA libraries, determined by RNA-sequencing.**

| **Sample** | **Raw**  **reads** | **Clean**  **reads** | **Clean**  **bases** | | **Error**  **rate(%)** | **Q20(%)** | **Q30(%)** | **GC_content**  **(%)** | **Total mapped(%)** | **Uniquely mapped(%)** |
| --- | --- | --- | --- | --- | --- | --- | --- | --- | --- | --- |
| W6_1 | 93,729,158 | 90,304,396 | 13.53 | 3.65 | | 97.88 | 94.10 | 47.86 | 95.49 | 90.16 |
| W6_2 | 105,587,052 | 101,965,806 | 15.27 | 3.43 | | 97.95 | 94.24 | 48.50 | 92.96 | 86.96 |
| W6_3 | 105,361,016 | 101,778,114 | 15.24 | 3.41 | | 97.94 | 94.24 | 50.05 | 94.30 | 87.07 |
| W14_1 | 111,668,572 | 106,942,618 | 16.02 | 4.22 | | 97.39 | 92.68 | 48.69 | 94.62 | 88.37 |
| W14_2 | 99,103,024 | 94,707,582 | 14.17 | 4.43 | | 97.37 | 92.65 | 46.20 | 95.04 | 89.74 |
| W14_3 | 107,542,426 | 102,587,480 | 15.37 | 4.61 | | 97.31 | 92.47 | 48.97 | 93.95 | 86.78 |
| W22_1 | 111,838,654 | 108,219,734 | 16.21 | 3.33 | | 97.92 | 94.19 | 51.52 | 94.71 | 84.27 |
| W22_2 | 116,176,278 | 112,568,564 | 16.86 | 3.11 | | 97.94 | 94.23 | 51.56 | 94.00 | 83.08 |
| W22_3 | 97,137,262 | 91,976,498 | 13.78 | 5.31 | | 97.15 | 92.11 | 47.87 | 95.59 | 90.02 |
| W30_1 | 113,865,916 | 109,898,532 | 16.45 | 3.48 | | 97.75 | 93.75 | 48.90 | 91.97 | 83.64 |
| W30_2 | 108,976,258 | 105,151,850 | 15.74 | 3.5 | | 97.75 | 93.75 | 47.94 | 93.30 | 86.68 |
| W30_3 | 116,860,112 | 112,729,594 | 16.88 | 3.53 | | 97.75 | 93.76 | 47.63 | 94.54 | 88.05 |

Abbreviations: W6_1, sample 1 of 6 weeks; W6_2, sample 2 of 6 weeks; W6_3, sample 3 of 6 weeks; W14_1, sample 1 of 14 weeks; W14_2, sample 2 of 14 weeks; W14_3, sample 3 of 14 weeks; W22_1, sample 1 of 22 weeks; W22_2, sample 2 of 22 weeks; W22_3, sample 3 of 22 weeks; W30_1, sample 1 of 30 weeks; W30_2, sample 2 of 30 weeks; W30_3, sample 3 of 30 weeks.
